# Seroepidemiology and assessment of risk factors for the spread of avian influenza in birds in two Nigerian states

**DOI:** 10.1002/vms3.73

**Published:** 2017-10-11

**Authors:** Musa Ibrahim Waziri, Paul A. Abdu, Lawal Sa'idu, Mohammed Bello

**Affiliations:** ^1^ Department of Veterinary Medicine Ahmadu Bello University Zaria Zaria Nigeria; ^2^ Veterinary Teaching Hospital Department of Veterinary Public Health and Preventive Medicine Ahmadu Bello University Zaria Zaria Nigeria; ^3^ Department of Public Health and Preventive Medicine Ahmadu Bello University Zaria Nigeria

**Keywords:** serology, epidemiology, risk factors, avian influenza, Nigeria

## Abstract

Despite modified stamping out eradication policy adopted in Nigeria, there was resurgence in 2015 of highly pathogenic avian influenza (HPAI) H5N1 with greater infectivity. A survey of the risk of spread of HPAI in two HPAI‐infected and ‐uninfected Nigerian states were studied. A cross‐sectional study to detect avian influenza (AI) H5 antibodies was conducted using haemagglutination inhibition (HI) test and enzyme‐linked immunosorbent assay (ELISA). A total of 950 birds’ sera were tested for AI H5 antibodies. Questionnaires were also administered to evaluate risks of AI spread in two states of Nigeria in 2013. AI H5 seroprevalence of 3% and 5% were obtained in Bauchi and Gombe states, respectively. Free flying and captive wild birds had 15% and 11% seroprevalence, respectively. Ninety‐two per cent AI awareness and 90% preparedness to report outbreaks of poultry diseases were recorded. Veterinary personnel, radio and television contributed 87% to HPAI awareness. Of the 10 risk categories evaluated, Gombe state had 3 moderate and 1 high risk of AI virus spread. Bauchi state recorded 5 moderate and 1 high risk of AI virus spread. Chi‐square analysis showed associations of altitude, temperature, rainfall and presence of live bird markets (LBMs) (*P* < 0.05) to AI seroprevalence. Odds ratio at 95% CI (1.313–6.333) indicated LBMs presence to be three times more likely to influence AI occurrence. HPAI H5N1 resurged in many states and occurred for the first time in Gombe state in 2015. Veterinary personnel, radio and television may be reliable in changing farmers’ attitudes to adopt good biosecurity practices.

## Introduction

A single‐stranded eight‐segmented RNA type A influenza virus of the family Orthomyxoviridae causes an acute and highly fatal disease in domestic and wild birds (Whiteman & Bickford 1989; Easterday *et al*. 1997; FAO 2007). Avian influenza (AI) initially occurred as a sporadic poultry disease worldwide (Easterday *et al*. 1997). However, AI H5N1 became a global concern in 1997 and 2003 when it caused human death in Hong Kong, China (Easterday *et al*. 1997; FAO 2007). The virus is known to mutate from low pathogenicity to high pathogenicity causing high mortality in poultry (FAO 2006). The synonyms ‘bird flu, fowl pest, fowl plague’ were therefore used to describe highly pathogenic avian influenza (HPAI) (Easterday *et al*. 1997; FAO 2007). This form is today a major emerging zoonosis which has a complex ecology and ability to infect other animal species (FAO 2006). Unlike the HPAI, the low‐pathogenic avian influenza (LPAI) is almost impossible to detect clinically; surveillance for this form is therefore dependent on sensitive and specific diagnostic procedures (Fouchier *et al*. 2006; OFFLU 2013). Transmission of avian influenza virus (AIV) could be directly through diseased or carrier birds or indirectly by contaminated personnel and formites to which the relative risk is dependent, on the degree of contact and ingestion of contaminated feed and water (Stegeman & Bouma 2004).

In Nigeria, the mechanism by which HPAI H5N1 virus spread to its neighbouring countries was believed to be environmentally influenced (Si *et al*. 2010). Routine AI surveillance has provided environmentally based data that informed critical prevention and control points in bird populations that subsequently reduced risk of human exposure (FAO 2007). An efficient HPAI virus surveillance and control therefore require the understanding of the mechanisms of the virus spread (Si *et al*. 2010). Serological surveillance specifically informed the status of AI in poultry and wild birds in many countries of the world (Adu *et al*. 1985; Shane & Durham 2006; FAO 2007; Capua & Alexander 2006; Teru *et al*. 2012; Wang *et al*. 2014). Measurement of a disease risk had provided effective and timely control efforts before disease outbreaks occurred. It also highlighted the efficiency of existing surveillance and demonstrated disease freedom in some countries (SPINAP 2011; OFFLU 2013). Countries that had outbreaks of HPAI suffered a fall in demand for poultry and poultry products for several years (Alexander 2000a). To this end, many countries became concerned on how best to prevent the introduction and spread of HPAI (Alexander 2000b). Therefore, risk assessment studies involving high‐risk areas should be intensified as a component of an early warning system. This study was conducted with a view to finding out the status of AI in one AI‐infected state where an active eradication policy had been going on for several years, and another immediate neighbouring state with apparent absence of AI.

## Materials and methods

### Study area

The study was carried out in Bauchi and Gombe states of north‐eastern Nigeria. Bauchi lies between latitudes 10° 10′ and 10° 33′ N and longitudes 9° 40′ and 10°13′ E. The vegetation is Sudan savannah type in the south and Sahel savannah in the central and northern regions. The state has 5 months of dry season (October–April). Some meteorological factors include annual rainfall record of 700–900 mm and humidity of 15–60% in the northern zone. In the central zone, 45–70% humidity and rainfall of 900–1300 mm are recorded. In the western zone, however, high humidity up to 90% and rainfall of about 1300 mm are common (BSADP 2003). Hot months are March and April, whereas cool months being February and August. The poultry population is over one million and comprises mainly rural poultry which are often bought and sold in live bird markets (BSADP 2003; NADIS 2006). This state had outbreaks of HPAI in 2006 and 2007 (NADIS 2006; Bello *et al*. 2008; Tesfai 2008). Gombe state is located entirely in the Sudan savannah between longitude 10° 45′ and 11° 45′ N and latitude 11° 15′ and 9° 30′ E. The state has a total annual rainfall of 850–1000 mm and two hot months (March and April). Humidity can be as low as 15% in the north, 25–50% in the central zone and up to 80% in southern zones. The poultry population is mainly rural poultry with few commercial (exotic) birds. Poultry are often traded live in many LBMs found scattered all over the two states. Despite closeness, free animal and human movements and trade in live birds, as of December 2014, Gombe state had not recorded HPAI outbreak (NADIS 2006, 2007; OIE 2013).

### Blood samples

Collection of blood from birds was conducted with the birds held and their wings extended to expose the brachial vein. A 21‐gauge needle attached to a 5‐mL syringe was inserted into the vein and a maximum of 2–3 mL of blood was collected. The blood was then transferred to a labelled plain blood collection tube and allowed to clot at room temperature. The clotted blood was then fractionated by centrifuging at 449 *g* for 5 min and the sera were transferred to labelled 2 mL containers and stored in a freezer at −20°C until analysed.

### Haemagglutination inhibition test

Antibody titre was determined by haemagglutination inhibition (HI) test as described by (Allan & Gough 1974; OIE 2005). The agglutination was positive in wells in which the RBCs streamed at the same rate as the control wells.

### Enzyme‐linked immunosorbent assay

Avian influenza H5 virus antibody ELISA kits were obtained from AffiniTech, Ltd. (USA) and IDVet (France), respectively. The test procedure was conducted according to the manufacturers’ instructions. The plates were read using dual wavelength microplate reader at 450 nm as primary filter and 650 nm as reference filter.

### Evaluation of risk factors for the spread of avian influenza and Newcastle disease

#### Questionnaire design and administration

To evaluate HPAI risks of spread based on expert opinions, a questionnaire approved by the Murdoch University Human Ethics Committee was modified and used for this study. It contained graded options for risk factors assessment which were categorized as negligible (0), very low (1), low (2), moderate (3), high (4) and very high (5), and then grouped into two risk groups (high = 4–5 and low = 0–3) (Keown & Hakstian 1973). Thirty questionnaires for each state were given to experienced poultry health workers (experts) to seek their opinions on the risk of AI spread into Bauchi and Gombe states out of which 24 and 22 questionnaires, respectively, were returned. To further evaluate risk factors associated with AI spread, 240 questionnaires were distributed to poultry farmers out of which 180 were duly completed and returned. Other sets of 10 questionnaires for each state targeting poultry sellers were also completed and returned.

#### Evaluation of risk factors associated with avian influenza seroprevalence

A high‐sensitivity global positioning system (GPS) device (*etrex* Garmin) was used to measure the longitude, latitude and the altitude of the study sites. Four villages from each senatorial district of the two states selected were grouped and assessed based on their locations on high (551–650 m), moderate (451–55 m) and low (300–450 m) altitudes. Some meteorological factors (rainfall, temperature and humidity) were obtained from Bauchi and Gombe states Agricultural Development Units and were grouped as high (901–1300 mm) and low (700–900 mm) rainfall records, moderate (27–32°C) and high (>32°C) temperature records, high (61–90%) and low (15–60%) humidity records. Records of the presence or absence of LBMs in the areas where samples were collected were also considered.

## Results

### Distribution and types of birds sampled for the study

**Figure 1:**
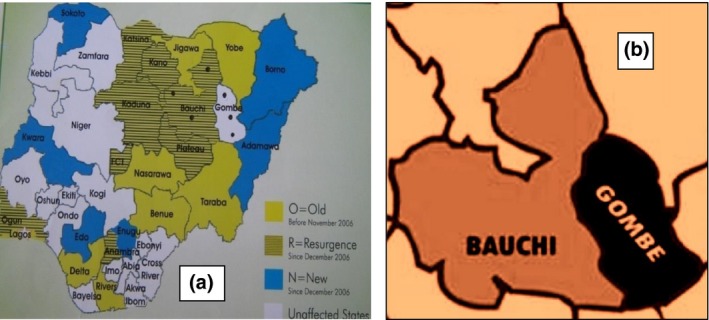
(a) Map of Nigeria showing the distribution of avian influenza outbreaks as of 2006/2007, the dots show locations of the study sites in HPAI‐affected (Bauchi) and HPAI‐unaffected (Gombe) states. (b) Positions of Bauch and Gombe states. **Source**: Avian influenza Media kit 2007.

A total of 1000 birds consisting of 910 domestic poultry, 21 semi‐domestic poultry and 69 wild bird species consisting of free flying and captive wild birds were sampled and tested as presented in Table 1. Figure 1a is the map of Nigeria showing the distribution of AI outbreaks as of 2006/2007, the dots indicate locations of the study sites in HPAI‐affected (Bauchi) and HPAI‐unaffected (Gombe) states, while Fig. 1b shows relative positions of Bauch and Gombe states.

**Table 1:** Distribution of semi‐domestic and wild bird types sampled for avian influenza and Newcastle disease antibodies and antigens in Bauchi and Gombe states, Nigeria

State	Type of wild bird	Scientific name	Native name (*Hausa*)	No. tested
	*Free flying (common name)*			
Bauchi	Rose‐ringed parakeet	*Psittacula krameri*	–	3
Bauchi and Gombe	Speckled/rock pigeon	*Columba guinea*	*Hasbiya*	6
Gombe	Bruce's green pigeon	*Treron waalia*	*Bili‐bili*	5
Bauchi	Senegal parrot	*Poicephalus senegalus*	*Aku*	4
Bauchi and Gombe	Cattle egret	*Bubulcus ibis*	*Belbela*	8
Bauchi and Gombe	Laughing dove	*Streptopelia senegalensis*	*Kurchiya*	6
	*Semi‐domestic species*			
Bauchi and Gombe	Moscovy duck	*Anas platyrhynchos*	*Agwagwa*	10
Bauchi and Gombe	Helmeted guinea fowl	*Numidia maleagris*	*Zabuwa*	11
	*Captive species*			
Gombe	African grey parrot	*Psittacus erithacus*	*Aku*	3
Bauchi	Four‐banded sandgrouse	*Pterocles quadrincinctus*	–	2
Bauchi	Black crowned crane	*Balearica pavonina*	*Gauraka*	3
Bauchi	Grey crowned crane	*Balearica regulorum*	*Gauraka*	1
Bauchi/Gombe	Feral pigeon	*Columba livia domestica*	*Tattabara*	10
Bauchi	Congo peacock	*Afropavo congensis*	*Dawisu*	2
Gombe	Canada goose	*Branta canadensis*	*Agwagwan ruwa*	5
Bauchi/Gombe	Ostrich	*Struthio camelus*	*Jimina*	4
Bauchi/Gombe	Little button quail	*Turnix sylvatica*	*Salwa*	7
Total				90

### Haemagglutination inhibition and enzyme‐linked immunosorbent assay test results

The results of sera tested for AI H5 antibodies using HI and ELISA in three local government areas (LGAs) of Bauchi and Gombe states are as presented in Table 2. The results indicated an overall seroprevalence of 3% obtained by each of the serological tests employed. The results further showed that the highest (8%) AI seroprevalence was obtained in Yamaltu/Deba LGA of Gombe state.

**Table 2:** Haemagglutination inhibition test and enzyme‐linked immunosorbent assay results of tested sera for H5 avian influenza antibodies in birds from three local government areas of Bauchi and Gombe states, Nigeria

State	Local government area	No. sera tested	HI (H5) positive (%)	Bird species	ELISA (H5) positive (%)	Bird species
Bauchi	Bauchi	166	4 (2)	cc lc wb, pg, tk	8 (5)	cc, lc, wb, tk,
	Katagum	164	8 (5)	lc, cc, tk	4 (2)	cc, tk
	Misau	170	4 (2)	cc, lc	2 (1)	lc
	Subtotal (prevalence)	500	16 (3)		14 (3)	
Gombe	Gombe	150	4 (3)	lc, dk, tk, gf	2 (1)	lc, dk, tk, gf,
	Kaltungo	150	6 (4)	lc, tk, cc	4 (3)	lc, tk, cc
	Yamaltu deba	150	12 (8)	lc, dk, gf	0 (0)	
	Subtotal (prevalence)	450	22 (5)	lc, dk, cc, gf	6 (1)	
	Grand total (prevalence)	950	38 (4)		20 (2)	

cc, commercial chicken; lc, local chicken; tk, turkey; pg, pigeon; gf, guinea fowl; wb, wild bird; dk, duck.

### Categories and assessment of risk factors for avian influenza spread

Experts’ opinions assessing AI risk categories and pathways of AI spread in the two states showed that 23 (77%) of the 30 scores in these regions had low AI risk of occurrence and 5 (17%) scores had moderate risk, while 2 (7%) had high risk of AI introduction into Bauchi state as presented in Table 3. In Gombe state, however, 25 scores (83%) had low‐risk and 1 (3%) had high‐risk category of AI introduction which was believed to be associated with trade in live birds. The 3 (10%) moderate risk scores assessed for AI introduction were associated with trade in live birds and trucks returning from infected areas as presented in Table 4. The risk assessment of poultry management practices revealed that most (60%) of the respondents kept poultry for income generation in Bauchi state. In Gombe state, however, 60% of respondents kept poultry for household consumption as well as for income generation. It was observed that 60% of farmers fed commercial feeds to their poultry and 20% provided poultry houses in Bauchi state. In Gombe state, however, 35% of the respondents fed commercial feeds to poultry and 9% of the respondents provided housing for poultry. However, it was observed that over 88% of the poultry were managed on free range in Gombe state. There was more (60%) and less (40%) of farmer control of water sources available to poultry flocks in Bauchi and Gombe states, respectively. More farmers (71%) access LBMs for the purpose of selling their poultry in Gombe than in Bauchi where 20% of the farmers were involved in this practice. However, poultry farmers in both states involved fowl sellers as middle men in selling their poultry. About 40% of poultry farmers used to return back unsold birds to farms and households in Bauchi state, while 67% of the poultry farmers had this practice in Gombe state as presented in Table 5.

**Table 3:** Twenty‐four experts opinions (*n* = 24) on risk categories of different pathways of introduction of avian influenza into north, central and south senatorial districts of Bauchi state, Nigeria

State	Bauchi								
District	North			Central			South		
Risk category	HR	LR	RC	HR	LR	RC	HR	LR	RC
Source of HPAI									
Frozen/dressed chicken from infected states	0	24	Low	0	24	Low	2	22	Low
Trade in live birds	3	21	Low	12	12	Moderate	22	2	High
Formites	6	18	Low	7	17	Low	20	4	High
Contaminated local surface water source	9	15	Low	5	19	Low	10	14	Low
Migratory wild birds	3	21	Low	3	21	Low	12	12	Moderate
Legal importation of birds	6	18	Low	2	22	Low	10	14	Low
Introduction of eggs from other places	9	15	Low	9	15	Low	12	12	Moderate
Returning trucks from infected areas	12	12	Moderate	11	13	Low	12	12	Moderate
Mutation (LPAI to HPAI)	4	20	Low	2	22	Low	8	16	Low

HR, high risk; LR, low risk; RC, risk category; LPAI, low‐pathogenic avian influenza; HPAI, highly pathogenic avian influenza.

**Table 4:** Twenty‐two experts opinions (*n* = 22) on the risk categories of different pathways of introduction of avian influenza into north, central and south regions of Gombe state, Nigeria

State	Gombe								
Region	North			Central			South		
Risk category	HR	LR	RC	HR	LR	RC	HR	LR	RC
Source of HPAI									
Frozen/dressed chicken from infected states	0	22	Low	0	22	Low	2	20	Low
Trade in live birds	3	19	Low	11	11	Moderate	20	2	High
Formites	6	16	Low	7	15	Low	9	13	Low
Contaminated local surface water source	9	13	Low	5	13	Low	10	12	Low
Migratory wild birds	3	19	Low	3	19	Low	4	18	Low
Legal importation of birds	6	16	Low	2	20	Low	10	12	Low
Introduction of eggs from other places	9	13	Low	9	13	Low	9	13	Low
Returning trucks from infected areas	11	11	Moderate	10	12	Low	11	11	Moderate
Mutation (LPAI to HPAI)	4	18	Low	2	20	Low	9	13	Low

HR, high risk; LR, low risk; RC, risk category; LPAI, low‐pathogenic avian influenza; HPAI, highly pathogenic avian influenza.

**Table 5:** Poultry husbandry practices by fowl sellers and poultry farmers in Bauchi (*n* = 90) and Gombe states (*n* = 90), Nigeria

	Bauchi		Gombe	
	Frequency	%	Frequency	%
Reasons for keeping poultry				
Eating	36	40	4	4.4
Selling	54	60	28	31.1
Both	18	20	60	66.7
Others	0	0	52	57.8
Feeding of poultry				
Commercial feed	54	60	32	35.6
Let them find	18	20	44	48.9
Both	18	20	0	0
Leftovers	0	0	64	71.1
Housing poultry				
Free room	54	60	80	88.9
Cage	18	20	8	8.7
Both	18	20	0	0
Drinking water source				
Own well	54	60	36	40
Community well	18	20	8	8.8
Surface water	0	0	48	53.3
Poultry trading				
Live bird market	18	20	64	71.1
Poultry trader	36	40	44	48.9
Households in same village	36	40	12	13.3
Return back unsold bird	36	40	60	66.7

### Poultry management and trading systems

Analysis of poultry marketing system indicated that fowl sellers did not entirely rely on live bird trade as a means of livelihood. It also indicated that 40–60% of respondents were engaged in the sale of different poultry species in the LBMs. About 30% of fowl sellers in Bauchi state obtained poultry within, and up to 40% obtained poultry outside their LGAs, respectively. However, 60% of fowl sellers in Gombe state obtained poultry within the same LGAs as presented in Table 5.

### Knowledge, awareness, practices and reporting of poultry diseases

The assessment of farmer's knowledge and willingness to report outbreaks of poultry diseases revealed that 100% and 84% of poultry farmers knew Newcastle disease as the most important poultry disease in Bauchi and Gombe states, respectively. On the other hand, 60% and 26% of poultry farmers in Bauchi and Gombe states, respectively, knew of salmonellosis but only 20% and 48% poultry farmers in Gombe state and Bauchi states, respectively, knew of other poultry diseases such as ectoparasitism and fowl pox; indeed, ectoparasitism (0%) and fowl pox (0%) were reported not to occur in Bauchi state. However, 100% and 84% of poultry farmers in Bauchi and Gombe states were willing to report outbreaks of poultry diseases to relevant authorities. HPAI was only recognized by 40% of Bauchi poultry farmers and reportedly was entirely unrecognized (0%) by Gombe state poultry farmers. It is also noted that 12% of poultry farmers were unwilling to report outbreaks of poultry diseases in Gombe state as presented in Table 6. Poultry farmers in Bauchi and Gombe states believed strongly (100%) and (80%), respectively, that the major sources of poultry disease to poultry flocks were contaminated feed and water. However, no farmer (0%) believed that wild birds could be a source of poultry disease outbreak. Bauchi state farmers were of the opinion that contact with sick birds (80%) and outbreaks from neighbouring farms (80%) would result into outbreaks in their farms, but in Gombe only 9% and 18% believed so as presented in Table 6. Assessment of HPAI showed that poultry farmers in Bauchi and Gombe states were 100% aware of HPAI. The means of awareness were mainly through veterinary personnel (80% and 93%), radio and television (80% and 93%), respectively. In Bauchi state, in addition, 60% and 80% sources of HPAI awareness were through community leaders and poultry traders, respectively. In contrast, however, 60% and 84% sources of HPAI awareness in Gombe state were due to daily newspapers and posters, respectively, as presented in Table 6.

**Table 6:** Farmers’ knowledge and reporting of common poultry diseases in Bauchi and Gombe states, Nigeria

	Bauchi		Gombe	
	Frequency	%	Frequency	%
Poultry disease				
Newcastle	90	100	76	84.4
Coccidiosis	36	40	12	13.3
Gumboro	18	20	20	22.2
Salmonellosis	54	60	24	26.7
Chronic respiratory disease	36	40	16	17.8
Ectoparasitism	0	0	20	22.2
Fowl pox	0	0	48	53.3
Highly pathogenic avian influenza	36	40	0	0.0
Reporting of poultry diseases				
Willing to report	90	100	76	84.4
Not willing to report	0	0	12	13.3
Report to relevant authority	90	100	76	84.4
Report to irrelevant authority	0	0	0	0

### Risk factors associated with the seroprevalence of avian influenza

The presence of LBMs in the study area was strongly associated (*P* = 0.004) with the seroprevalence of AI. Also low level of rainfall was associated (*P* = 0.033) with AI seroprevalence. Temperature was as well associated (*P* = 0.033) with AI seroprevalence. AI seroprevalence was also strongly associated (0.000) with low altitude in this study as presented in Table 4. Odds ratio (OR) at 95% CI (1.313–6.333) showed about three times likelihood of AI seroprevalence in regions where LBMs were located. Rainfall as a risk factor was assessed at 95% (1.051–4.832), temperature was also assessed at 95% CI (1.018–4.760) and were each found to influence AI seroprevalence about two times (odds ratios‐2.254 and 2.234 respectively). There were no significant associations of states and senatorial districts to the seroprevalence of AI (*P* > 0.05) as presented in Table 7.

**Table 7:** Analysis of risk factors associated with avian influenza seroprevalence in Bauchi and Gombe states, Nigeria

Category	Risk factor	No. of sera tested	No. of sera positive for AI antibody	% positive	χ^2^	df	*P*‐value	Odds ratio
State	Bauchi	240	15	6.2	0.199	1	0.656	1.190
	Gombe	245	13	5.3				
District	Bauchi North	72	6	8.3	2.879	5	0.719	1^a^
	Bauchi South	83	6	7.2				0.857
	Bauchi Central	85	3	3.5				0.402
	Gombe North	85	3	3.5				0.402
	Gombe South	75	5	6.7				0.786
	Gombe Central	85	5	5.9				0.689
Altitude	High (551–650 m)	130	8	6.2	0.901	2	0.000	1^a^
	Moderate (451–550 m)	132	5	3.8				0.660
	Low (300–450 m)	223	5	5.8				0.944
Rainfall	Low (700‐900 mm)	193	17	8.8	4.563	1	0.033	2.254
	High (901–1300 mm)	292	12	4.1				
Temperature	Moderate (27–32°C)	178	16	9.0	4.530	1	0.033	2.234
	High (>32°C)	307	13	4.2				
Humidity	Low (15–60%)	337	18	5.3	0.800	1	0.371	0.703
	High (61–90%)	148	11	7.4				
Live bird market	Present	165	17	10.3	8.316	1	0.004	2.948
	Absent	320	12	3.8				

a Point of comparison.

## Discussion

An efficient AI control system requires a greater understanding of the mechanism responsible for the spread of AIV (Si *et al*. 2010). Two hypotheses suggest roles of wild birds and human activities in poultry production and trading systems in aiding the global spread of HPAI in many countries of the world (FAO 2006; Alexander 2000a). Wild birds pose a potential risk to biosecurity because they can transfer pathogens into poultry farms. Therefore, disease surveillance in wild birds and poultry has become unavoidable in order to avert outbreaks of avian diseases. Results of serological investigation carried out in this study showed that apparently healthy domestic and wild birds were exposed to H5 AIV in Bauchi and Gombe states, Nigeria. This appears crucial because the Nigerian Government embarked upon HPAI‐modified stamping out eradication policy since 2006 (NADIS 2006). This policy recorded an apparent absence of HPAI outbreaks in Nigeria over 7 years (2008–2014). Yet serological surveillance of AI reliably indicated how domestic poultry, free living and captive wild birds could be exposed to AIV in many countries. These findings further alerted the attention of livestock policy makers to reconsider wider threats of AI incursion. This is important because any HPAI‐infected country is automatically banned from participation in international trade of live birds and birds’ products (FAO 2006; OIE 2015), and Nigeria trades in live poultry and poultry products with many countries, and therefore must adhere to world trade regulations on poultry trade (EMPRES 2015).

In this study, AI H5 seroprevalence of 3% from each state was obtained using HI and ELISA. The two serological tests are sensitive and specific (Jensen *et al*. 2013), indicating that birds in the study areas were actually exposed to AI H5 as vaccination of poultry against AI in Nigeria was prohibited since 2006 (NADIS 2006; Jensen *et al*. 2013). Serological surveillance offers a relatively fast, inexpensive and practical way to determine viruses circulating in the avian species and contributes significantly to monitoring AI‐free status (Cattoli & Capua 2007). To date, almost all field infections with HPAI viruses have been of HA subtypes H5 and H7, and under certain conditions, the H5 and H7 LPAI could circulate in different bird species to enable mutations to HPAI viruses. Hence, all HPAI viruses and LPAI viruses of subtypes H5 and H7 in poultry are termed notifiable to OIE (2015).

In Gombe state, the highest site‐specific prevalence was in Kwadon which happened to be a satellite town of Gombe. Kwadon is located in a Fadama area with fairly thick vegetation cover and had the highest density of commercial poultry activities and a major weekly LBM. These features were documented among others as some of the most important risk factors for AI occurrence worldwide (FAO 2006; Fasina *et al*. 2007; Han *et al*. 2014). Earlier, high‐risk and poor biosecurity practices likely to support AI introduction and spread were reported among poultry farmers in Bauchi and Gombe states (Musa *et al*. 2010). Incidentally, Gombe state recently reported AI outbreaks for the first time. The index case was from Kwadon (OIE 2015).

In this study, we detected AI antibodies in local chicken, duck and black crowned crane. Water fowls including ducks have been tagged common reservoirs of AIVs (FAO 2007). Devastating outbreaks of AI and ND have been reported in crowned cranes in many places. Also, loss of habitat and illegal crowned crane trade has made this species of wild bird endangered (FAO 2007; Bello *et al*. 2008). In the wild, crowned cranes undergo seasonal movements and congregate at periphery of water bodies (FAO 2007), in Nigeria they are kept as pets by rich individuals (Bello *et al*. 2008). These attributes make crowned cranes potential source of AI transmission at aquatic habitats, to poultry farms and as possible source of human exposure to HPAI especially where they are kept as pets as seen in one of the study areas.

Risk analysis has been used in many countries to identify and communicate to stakeholders specific disease risks based on many criteria including experts’ opinions (FAO 2014). This study assessed a known HPAI‐infected state (Bauchi) in 2006–2007 and HPAI‐free state (Gombe) as in December 2014 (NADIS 2006; Bello *et al*. 2008; Tesfai 2008). It was observed in this study that the knowledge of Newcastle disease in Bauchi (100%) and Gombe (84%) was very high, indicating that ND was probably the most encountered disease of poultry in these states. Unfortunately, farmers’ knowledge of HPAI was very poor, which would affect HPAI urgent reporting as clinical manifestations of AI and ND are similar (FAO 2006). Unlike Gombe, Bauchi poultry farmers experienced outbreaks of HPAI and could recognize that sick birds could be a serious source of infections to healthy flocks and also infected neighbouring farms could transmit poultry diseases to uninfected farms. However, poultry farmers from both states had very poor (0–20%) knowledge of the role of formites in poultry disease transmission. This is of concern as Wang *et al*. (2008) isolated AIV in bird cages in China. Moreover, clothing, equipment, crates and vehicles were reported to transmit NDV (Alexander & Allan 1974), and AIV was reported to survive in infected birds’ feathers for up to 18 days (Purchase 1931). Again both farmers underestimated (0%) the role of wild birds in poultry disease transmission. In many countries of the world, especially in China, Russia and the United Kingdom, however, migratory wild birds were linked to HPAI outbreaks (Chen *et al*. 2006), and Artois *et al*. (2009) reported likelihood of free ranging poultry exposure to AIV.

From this study, it was clear that veterinary personnel, radio and television contributed most to HPAI awareness in the two states. This therefore suggests that advocacy using veterinary personnel, radio and television could be very effective in creating awareness of HPAI and other poultry diseases. This is very necessary because any disease risk analysis process should involve risk identification, assessment, management and most importantly risk communication (FAO 2012; EMPRES 2015; SPINAP 2011). This study further showed that most poultry owners in Gombe state sold live birds during weekly market days. Poultry buyers from villages and urban markets in these states often buy and transport these birds to re‐sale in many neighbouring cities. This attitude was also reported by Leigh *et al*. (2010) in some states of Nigeria. Unlike in Gombe state, inter‐local government live bird trading was common in Bauchi state which could facilitate the spread poultry pathogens. It was earlier reported that many places along the major route of inter‐state movement of live birds from south to northern Nigeria recorded most outbreaks of HPAI in 2006 (Fasina *et al*. 2007). Ekong *et al*. (2012) in their spatiotemporal analysis of HPAI H5N1 spread in 2006–2008 also reported local and long distance spread in Nigeria. Commercial poultry in Nigeria are mainly moved from southern Nigeria to the north, while live local chickens and guinea fowls were moved from the north to the south. This movement network might have played a significant role in poultry disease spread in Nigeria (EMPRES 2015). The study of live bird marketing reported in this study showed that multiple species of birds from several places were temporarily held in cages in LBMs before being sold out. Also, local poultry of different species were kept on free range in most households in the study area. Abdu *et al*. (2013) reported same practice of keeping more than one species of poultry and other livestock species in farms and households in Kaduna state. Bavinck *et al*. (2009) in his study showed that keeping multiple species of birds in poultry farms had increased risk of poultry disease outbreaks. Backyard poultry was also reported to be a major source of spread and persistent of HPAI H5N1 in Asia (Tiensin *et al*. 2005). Fasina *et al*. (2007) in his spatial factor risk considerations for HPAI spread in Nigeria identified LBMs to be responsible for long distance spread HPAI in Nigeria.

Some meteorological factors like rainfall, humidity and altitude were in this study found to be associated with the seroprevalence of AI. Lowen *et al*. (2007) experimentally showed that aerosol spread of AIV was dependent on both ambient relative humidity (RH) and temperature (T). He reported that low RH (20–35%) favoured virus transmission, while high RH (80%) retarded transmission. Greater frequency of AIV transmission occurred at low temperature (5%) than at 20°C. They concluded that low RH and low temperature might be contributing to seasonality of influenza infections. The association of low altitude and low rainfall with AI prevalence observed in this study has not been previously documented in Nigeria. However, low altitudes of 0–100 m were reported to be associated with a high risk of HPAI H5N1 occurrence in China (Martin *et al*. 2011). Si *et al*. (2010) showed high correlation between HPAI occurrence and lower elevations. The relationship of AI seroprevalence to low altitude reported in our study could be attributed to the fact that most low‐altitude areas, especially in the studied locations, were liable to periodic flooding in which poultry farms located at lower altitudes could be liable to periodic run‐offs by rainfall and thus could feature as a source of virus transmission. Brown *et al*. (2007) and Martin *et al*. (2011) reported that AIV was able to persist in water and that low altitude facilitated the transmission of the virus indirectly excreted into the environment so that surface water transmitted the virus to poultry farms located downstream. Si *et al*. (2010) also concluded that lower elevations and reduced rainfall correlated very well with HPAI occurrence.

## Source of funding

The study was a postgraduate research that was mainly self‐financed and partly funded by the Ahmadu Bello University, Zaria, Nigeria.

## Conflict of interest

There was no conflict of interest encountered during the conduct of this research as well as documenting and publishing these findings.

## Ethics statement

This study was part of a postgraduate research conducted in the postgraduate school of Ahmadu Bello University, Zaria, Nigeria. Until recently, there was no functional ethical committee on animal use in the university. Proposal to humanely conduct the study was presented to and approved by the Faculty of Veterinary Medicine Postgraduate Committee.

## Contribution

This study indicated the need for continuous AI risk based surveillance in infected as well as free areas for possible implementation of timely control measures.
